# Quality of histone modification antibodies undermines chromatin biology research

**DOI:** 10.12688/f1000research.7265.2

**Published:** 2015-11-24

**Authors:** Goran Kungulovski, Albert Jeltsch

**Affiliations:** 1 Institute of Biochemistry, Stuttgart University, Stuttgart, 70569, Germany

**Keywords:** histone modifications, antibodies, recombinant proteins, quality control

## Abstract

Histone post-translational modification (PTM) antibodies are essential research reagents in chromatin biology. However, they suffer from variable properties and insufficient documentation of quality. Antibody manufacturers and vendors should provide detailed lot-specific documentation of quality, rendering further quality checks by end-customers unnecessary. A shift from polyclonal antibodies towards sustainable reagents like monoclonal or recombinant antibodies or histone binding domains would help to improve the reproducibility of experimental work in this field.

The lack of reproducibility is widely recognized as a serious issue in contemporary research (see ( Buck, 2015; Freedman & Inglese, 2014; Freedman *et al.*, 2015; McNutt, 2014a; McNutt, 2014b) and the Nature special “Challenges in irreproducible research” April 2, 2013). In molecular biology, the quality of antibodies has been identified and highlighted as one of the most recurring stumbling blocks that undermine the quality and validity of experimental results ( Baker, 2015; Bordeaux *et al.*, 2010; Bradbury & Plückthun, 2015a; Bradbury & Plückthun, 2015b). This issue is even more pervasive in the field of molecular epigenetics and chromatin biology, where antibodies for various types of histone post translational modifications (PTMs) have been single-handedly used to translate the language of histone modifications into experimentally observable properties. Because of this, most of what we know about the distribution, role and function of histone modifications so far has been passed through an antibody as essential mediator.

Raising a specific histone modification antibody is not a trivial task; this is mostly due to the hypermodified state of the histone tail, coupled with the minute size and the chemical relatedness of many histone modifications and similarities in the amino acid sequence of the modified residues. The antibody has to be able to discriminate between the unmodified and the modified state of the targeted amino acid residue, as well as between different forms of modifications (e.g. acetylations of different lysine residues, mono-, di- and trimethylation of lysine residues, or symmetric and asymmetric methylation of arginine residues). Moreover, the presence of an adjacent modification might prevent binding of an antibody to the target modification, causing false negative results. In addition, the antibody should bind the modified amino acid residue only at defined modification sites on the target protein, which implies that not only the modification but also the amino acid sequence must be recognized. This is particularly difficult for some histone modifications such as methylation or acetylation of H3K9 and H3K27 which occur within an identical amino acid context (ARKS motif) and make the readout of the target peptide sequence outside of this central motif vital as well.

In spite of the intricate task of producing histone modification antibodies and their crucial role in chromatin biology, surprisingly, they remain insufficiently characterized. In line with this, numerous scientific groups have alarmingly raised concerns about the promiscuous behavior of some histone modification antibodies and undocumented effects of secondary modifications ( Bock *et al.*, 2011; Egelhofer *et al.*, 2011; Hattori *et al.*, 2013; Kungulovski *et al.*, 2014; Nishikori *et al.*, 2012; Rothbart *et al.*, 2015). As mentioned above, the situation in chromatin biology is exceptional, because of the role of histone PTM antibodies as the sole research tool in this field. As a consequence, elaborate quality control criteria for histone PTM antibodies were put forward to ensure the integrity of research ( Egelhofer *et al.*, 2011; Kungulovski *et al.*, 2015; Landt *et al.*, 2012). To increase transparency, at least two databases for deposition of antibody quality data from researchers were put in place ( http://compbio.med.harvard.edu/antibodies/; http://www.histoneantibodies.com/) ( Egelhofer *et al.*, 2011; Rothbart *et al.*, 2015). However, in spite of being heroic attempts, these and similar databases have only a limited value, because most of the antibodies used in chromatin biology are polyclonal, and lab experience over the last years has demonstrated that the specificity data obtained for one batch of antibody do not necessarily reflect the properties of another one ( Kungulovski *et al.*, 2014), a caveat which is still often ignored by naïve end-users. Related to this the practice of some antibody manufactures of retaining catalog numbers for new polyclonal antibody lots is unacceptable and misleading as well as the practice to use historical data sheets for antibodies to which they do not apply ( Voskuil, 2014).

The necessary quality control steps for histone modification antibodies ( Egelhofer *et al.*, 2011; Kungulovski *et al.*, 2015; Landt *et al.*, 2012) currently burden the individual antibody user with high costs and workload. Given that antibodies are expensive reagents, which are of no use without appropriate quality documentation, these efforts must be redirected from the end-customer to the manufacturers of antibodies. Herein, we urgently ask the vendors and manufacturers of antibodies to provide the necessary product sheets for all types of antibodies on a regular basis, including quality control documentation for each batch of polyclonal and each catalog number of recombinant or monoclonal antibodies. Results of the following validation tests must be provided to enable the end-user finding the information, which is particularly relevant for the intended application of an antibody:

1. Combinatorial profiling of specificity with peptide arrays or similar high-throughput methods. If possible, profiling of specificity with recombinant and semisynthetic nucleosomes harboring different modifications.
2. Western blot results with native (as positive control) and recombinant histones (as negative control).
3. Western blot results with native histones or nuclear extracts from cells where the responsible histone modifying enzyme has been deleted or depleted (mammalian cells) or mutant histones (yeast).
4. Reproducibility of ChIP-seq data and high correlation with similar validated ChIP-seq datasets.

As proposed by others ( Bradbury & Plückthun, 2015a; Bradbury & Plückthun, 2015b) end-users should consider boycotting companies not complying with this demand, or at least stay away from products lacking a proper lot-specific documentation. While one may expect that better quality control will increase the prices of commercial antibodies, end-customers will not be forced to conduct their own quality control and they will not waste money for non-functional antibodies, so that the overall final costs may not be much higher. Moreover, the value of the obtained data will increase massively with better antibody validation.

The batch-to-batch variability of critical properties like cross reactivity or inhibition by secondary marks makes the application of polyclonal antibodies intrinsically unsustainable, because experiments cannot be reproduced after the corresponding batch of an antibody is sold out. As a consequence of this, rigorously speaking, large data sets in chromatin biology exist in a “grey” area outside of natural science, since it is impossible to repeat the underlying experiments. In a long-term perspective, a shift away from polyclonal antibodies towards alternative reagents, which can be produced at constant quality, would help to reduce the necessary financial and workload efforts associated with quality control of polyclonal antibodies and ensure sustainability ( Bradbury & Plückthun, 2015a; Bradbury & Plückthun, 2015b). This applies to high quality monoclonal antibodies, recombinant antibodies ( Hattori *et al.*, 2013) or analogous recombinant reading domains ( Kungulovski *et al.*, 2014). This will not only help to reduce costs in chromatin research in the long run (once obtained, the documentation will be valid for all lots) but also help to standardize the affinity reagents used and ease the lab-to-lab comparison of data. The recombinant reagents are particularly promising, because their sequences can be published, which ensures full transparency and reproducibility. Of note, in chromatin biology native reading domains designed by nature to specifically recognize relevant histone PTM marks are available as an alternative to antibodies ( Kungulovski *et al.*, 2014), which is an advantage over other fields, where recombinant production of antibodies is the only technical solution to the issue of reproducible performance and long term availability of these essential research reagents.
