# Supplementary material for: Quality of histone modification antibodies undermines chromatin biology research
Source: F1000Res. 2015 Nov 24;4:1160. Originally published 2015 Oct 28. [Version 2] doi: 10.12688/f1000research.7265.2 (PMC4706057; doi:10.12688/f1000research.7265.2)
Supplement: Peer review history

# Peer review: Referee response for version 2

**DOI:** 10.5256/f1000research.7991.r11772

**Published:** 2016-01-18

**Authors:** Shohei Koide

**Affiliations:** 1 Department of Biochemistry and Molecular Biology, Knapp Center for Biomedical Discovery, University of Chicago, Chicago, IL, USA

The authors concentrate on their core message for better characterization and documentation and for shifting toward renewable reagents. It is important to note that more characterization would be better but demanding the inclusion of assays of limited predictive power would increase the cost of antibodies without substantially benefiting the end users.

"Moreover, we are not convinced that the development of special conditions by each lab is an advisable development, because in many cases these may not be fully documented which - again - would undermine reproducibility. While it is self-evident that the researchers are ultimately responsible for the validity of all their results, lab internal quality certifications in our view are not the best way to proceed in an ever growing experimental field."

This is not what meant in my review. Instead, I mean that what an end user studies would always represents novel conditions (different cells, different culture conditions, different number of cells, etc.). So, strictly speaking, there is no guarantee that even a highly validated antibody functions in the actual experiment. The field would benefit greatly by establishing better internal controls that are included in each experiment, particularly for ChIP.

I have read this submission. I believe that I have an appropriate level of expertise to confirm that it is of an acceptable scientific standard.

# Peer review: Referee response for version 2

**DOI:** 10.5256/f1000research.7991.r11774

**Published:** 2016-01-04

**Authors:** Andrew Bradbury

**Affiliations:** 1 Bioscience Division, Los Alamos National Laboratory, Los Alamos, NM, USA

The authors have addressed my concerns

I have read this submission. I believe that I have an appropriate level of expertise to confirm that it is of an acceptable scientific standard.

# Peer review: Referee response for version 1

**DOI:** 10.5256/f1000research.7827.r10962

**Published:** 2015-11-19

**Authors:** Shohei Koide

**Affiliations:** 1 Department of Biochemistry and Molecular Biology, Knapp Center for Biomedical Discovery, University of Chicago, Chicago, IL, USA

This paper succinctly reviews antibody-related problems that have been widely recognized in the biological and biomedical community, in a specific context of anti-histone posttranslational modification (PTM) antibodies and their uses in epigenetic research. The authors correctly emphasize the large negative impact of the batch-to-batch variation of anti-histone PTM antibodies and its consequence ('large data sets in chromatin biology exist in a “grey” area outside of natural science, since it is impossible to repeat the underlying experiments.'). I generally agree with the current challenges described in this paper, but it could be improved by addressing the following points.

An important omission in the paper is the limited recognition by users of potentially large effects of the mismatch between validation methods and real applications. We can perhaps agree that antibodies should ideally be validated in a manner that closely mimics how it is used in the actual application. Validation methods can be divided into two general types based on their formats: one in which antibodies are immobilized and captured antigens are detected ("immunoprecipation (IP) type") and the other in which antigens are immobilized and captured antibodies are detected ("blotting type"). Peptide arrays and Western blotting fall into the latter blotting-type validation methods, in which peptides or denatured proteins are localized, often at high density, on a solid support and binding of dilute antibody samples is detected. To state the obvious, IP-type methods are suited for validating antibodies for IP-type applications (IP, ChIP), whereas blotting-type methods are suited for validating antibodies for blotting-type applications (Western, immunostaining).

Potential problems arise when the validation format is different from the application format. It is not easy to predict whether antibodies validated using blotting-type methods perform well in IP-type applications such as ChIP, and vise versa. Egelhofer *et al*. (2011) reported that more than 20% of antibodies that have been validated to be specific in peptide blots still fail in ChIP experiments. In typical IP applications where an antibody is immobilized on a solid support, antibody affinity is a critical parameter. In contrast, affinity is not critical in blotting type applications, because the bivalent format of the conventional antibody (i.e. two antigen-binding sites per molecule) helps boost binding (the so-called avidity effect). Indeed, it has been found that an antibody that looked good on peptide arrays performed poorly in IP and conversely another antibody that did not look good on peptide arrays performed exceedingly well in IP (Nishikori *et al., *2012). Similarly, an antibody (Active Motif 39156) performed well in IP validation using semi-synthetic nucleosomes ("IceChIP"), although it appeared cross-reactive in peptide-array validation and in Western blotting (Rothbart *et al.*, 2015). Further complications arise from the fact that spot intensities in array-type experiments are not quantitatively correlated with the strengths of the measured interactions (see, for examples, Stiffler *et al*., 2006; Hause *et al. *2012).

Another omission is that validation and actual results depend on experimental conditions and accordingly an antibody validated under one set of conditions may not perform as well under a different set of conditions. Practitioners of immunoblotting are all familiar with the need for "optimizing" conditions for their own experiments. Similar optimizations are needed for other types of applications for which desired outcomes are less obvious. Furthermore, the abundance of the antigen of interest relative to off targets influences the outcome. Even a highly selective and potent antibody may not sufficiently enrich extremely rare antigens. Accordingly, for IP-type applications, mass spectroscopy-based validation using IP with input materials similar to those used in actual experiments (Peach *et al*., 2012; Hattori *et al., *2013; Marcon *et al. *2015) and IP of semi-synthetic nucleosomes (Grzybowski *et al., *2015) are better suited as validation methods.

This paper should emphasize more that the end user must critically evaluate limitations of validation methods caused by format mismatches and/or variations in experimental conditions. I do not agree with this paper's recommendations that a single set of information be provided with any antibody regardless of its intended use.

I have read this submission. I believe that I have an appropriate level of expertise to confirm that it is of an acceptable scientific standard, however I have significant reservations, as outlined above.

## References

[1] EgelhoferTAMinodaAKlugmanSLeeKKolasinska-ZwierzPAlekseyenkoAACheungMSDayDSGadelSGorchakovAAGuTKharchenkoPVKuanSLatorreILinder-BassoDLuuYNgoQPerryMRechtsteinerARiddleNCSchwartzYBShanowerGAVielleAAhringerJElginSCKurodaMIPirrottaVRenBStromeSParkPJKarpenGHHawkinsRDLiebJD: An assessment of histone-modification antibody quality. *Nat Struct Mol Biol*.2011;18(1) : 10.1038/nsmb.1972 91-3 10.1038/nsmb.1972 21131980PMC3017233

[2] NishikoriSHattoriTFuchsSMYasuiNWojcikJKoideAStrahlBDKoideS: Broad ranges of affinity and specificity of anti-histone antibodies revealed by a quantitative peptide immunoprecipitation assay. *J Mol Biol*.2012;424(5) : 10.1016/j.jmb.2012.09.022 391-9 10.1016/j.jmb.2012.09.022 23041298PMC3502729

[3] RothbartSBDicksonBMRaabJRGrzybowskiATKrajewskiKGuoAHShanleEKJosefowiczSZFuchsSMAllisCDMagnusonTRRuthenburgAJStrahlBD: An Interactive Database for the Assessment of Histone Antibody Specificity. *Mol Cell*.2015;59(3) : 10.1016/j.molcel.2015.06.022 502-11 10.1016/j.molcel.2015.06.022 26212453PMC4530063

[4] StifflerMAGrantcharovaVPSeveckaMMacBeathG: Uncovering quantitative protein interaction networks for mouse PDZ domains using protein microarrays. *J Am Chem Soc*.2006;128(17) : 10.1021/ja060943h 5913-22 10.1021/ja060943h 16637659PMC2533859

[5] HauseRJLeungKKBarkingeJLCiaccioMFChuuCPJonesRB: Comprehensive binary interaction mapping of SH2 domains via fluorescence polarization reveals novel functional diversification of ErbB receptors. *PLoS One*.2012;7(9) : 10.1371/journal.pone.0044471 e44471 10.1371/journal.pone.0044471 22973453PMC3433420

[6] PeachSERudominELUdeshiNDCarrSAJaffeJD: Quantitative assessment of chromatin immunoprecipitation grade antibodies directed against histone modifications reveals patterns of co-occurring marks on histone protein molecules. *Mol Cell Proteomics*.2012;11(5) : 10.1074/mcp.M111.015941 128-37 10.1074/mcp.M111.015941 22442256PMC3418838

[7] HattoriTTaftJMSwistKMLuoHWittHSlatteryMKoideARuthenburgAJKrajewskiKStrahlBDWhiteKPFarnhamPJZhaoYKoideS: Recombinant antibodies to histone post-translational modifications. *Nat Methods*.2013;10(10) : 10.1038/nmeth.2605 992-5 10.1038/nmeth.2605 23955773PMC3828030

[8] MarconEJainHBhattacharyaAGuoHPhanseSPuSByramGCollinsBCDowdellEFennerMGuoXHutchinsonAKennedyJJKrastinsBLarsenBLinZYLopezMFLoppnauPMierschSNguyenTOlsenJBPaduchMRavichandranMSeitovaAVadaliGVogelsangMSWhiteakerJRZhongGZhongNZhaoLAebersoldRArrowsmithCHEmiliAFrappierLGingrasACGstaigerMPaulovichAGKoideSKossiakoffAASidhuSSWodakSJGräslundSGreenblattJFEdwardsAM: Assessment of a method to characterize antibody selectivity and specificity for use in immunoprecipitation. *Nat Methods*.2015;12(8) : 10.1038/nmeth.3472 725-31 10.1038/nmeth.3472 26121405

[9] GrzybowskiATChenZRuthenburgAJ: Calibrating ChIP-Seq with Nucleosomal Internal Standards to Measure Histone Modification Density Genome Wide. *Mol Cell*.2015;58(5) : 10.1016/j.molcel.2015.04.022 886-99 10.1016/j.molcel.2015.04.022 26004229PMC4458216

# Response

**Published:** 2015-11-24

**Authors:** Albert Jeltsch

**Affiliations:** Uni Stuttgart, Germany

We agree with the general conclusions of this reviewer that validation methods and protocols need to be adjusted to the planned applications. However, it should be noticed that the main aim of this short opinion paper was to further raise concerns and contribute to a move towards better documentation and sustainable reagents in chromatin research. The current state is that antibody documentation is widely insufficient and the reagents are often not sustainable. We propose to improve this situation step by step. It would be an important advance for the field to have a panel of standardized quality documentation data available for each antibody lot. This includes data in different formats, which will allow users to select the most relevant information for their experiments. To take up the point of this reviewer, we included a hint that validation methods must reflect the intended application of antibodies.

Defining the best format of quality checks will be an ongoing challenge for the entire field. We already expressed a preferences for the application of recombinant modified nucleosomes as validation method in chromatin biology in the original version of the manuscript. Another very important improvement for the field would be to have more recombinant affinity reagents, which are fully sustainable.

However, we like to mention that we are not fully convinced by the statement that the differences in technical formats (blotting vs. pull-down) is the only or main reason of differences in antibody performance between certain assays. One additional, very critical, difference is that short peptides are used in many validation assays (for practical reasons) but real applications deal with the pull-down of full histone tails. Moreover, we are not convinced that the development of special conditions by each lab is an advisable development, because in many cases these may not be fully documented which - again - would undermine reproducibility. While it is self-evident that the researchers are ultimately responsible for the validity of all their results, lab internal quality certifications in our view are not the best way to proceed in an ever growing experimental field.

# Peer review: Referee response for version 1

**DOI:** 10.5256/f1000research.7827.r10993

**Published:** 2015-11-03

**Authors:** Scott B. Rothbart

**Affiliations:** 1 Center for Epigenetics, Van Andel Institute, Grand Rapids, MI, USA

Antibody reliability in biomedical research is of utmost importance. The quality of these reagents in chromatin biology applications is of particular concern given their position as essential tools for most techniques characterizing the cellular abundance and genomic distribution of histone post-translational modifications (PTMs).

I agree with the Kungulovski and Jeltsch that increased accountability needs to be demanded from companies who sell histone PTM antibodies, and their four recommended quality control measures are reasonable expectations. In addition, it should also be noted that the practice of retaining catalog numbers for new polyclonal antibody lots is unacceptable and misleading.

Moreover, and particularly in light of the increased awareness of antibody concerns in the field, experimentalists and epigenome consortium leaders (e.g., ENCODE, BLUEPRINT) should be more rigorous in their own evaluation of histone PTM antibodies when choosing a reagent for their study. Antibody specificity data and lot numbers used should also be standard requests from journal editors and reviewers.

Accountability clearly needs to come from all parties if we are to continue benefiting from the use of these affinity tools in chromatin research.

I have read this submission. I believe that I have an appropriate level of expertise to confirm that it is of an acceptable scientific standard.

# Response

**Published:** 2015-11-12

**Authors:** Albert Jeltsch

**Affiliations:** Uni Stuttgart, Germany

- *“I agree with the Kungulovski and Jeltsch that increased accountability needs to be demanded from companies who sell histone PTM antibodies, and their four recommended quality control measures are reasonable expectations. In addition, it should also be noted that the practice of retaining catalog numbers for new polyclonal antibody lots is unacceptable and misleading.”*
  Reply: We have added the sentence to the paper “that the practice of retaining catalog numbers for new polyclonal antibody lots is unacceptable and misleading”. Thanks a lot for this helpful suggestion.
- *“Moreover, and particularly in light of the increased awareness of antibody concerns in the field, experimentalists and epigenome consortium leaders (e.g., ENCODE, BLUEPRINT) should be more rigorous in their own evaluation of histone PTM antibodies when choosing a reagent for their study. Antibody specificity data and lot numbers used should also be standard requests from journal editors and reviewers. Accountability clearly needs to come from all parties if we are to continue benefiting from the use of these affinity tools in chromatin research.”*
  Reply: We like to mention, that in our view more responsibility in structured product documentation lies at the side of the supplier. Quality checks done by end-customers are an emergency action, but they will not solve the problem of long-term and lab-to-lab reproducibility. Also they put all financial pressure on the end customer or a product, which is not common practice in other parts of the economy.

# Peer review: Referee response for version 1

**DOI:** 10.5256/f1000research.7827.r10961

**Published:** 2015-11-02

**Authors:** Andrew Bradbury

**Affiliations:** 1 Bioscience Division, Los Alamos National Laboratory, Los Alamos, NM, USA

Paragraph 2 should also indicate that antibodies purportedly recognizing PTMs at specific sites, need to have their recognition specificity also tested against the same PTM at different sites, in the same protein, or others, and with the same core sequence or others. Many so-called specific phosphotyrosine antibodies actually recognize the phosphotyrosine modification independently of its sequence context.

"In spite of the intricate task of producing histone modification antibodies and their crucial role in chromatin biology, surprisingly, they remain to be insufficiently characterized" should be changed to "In spite of the intricate task of producing histone modification antibodies and their crucial role in chromatin biology, surprisingly, they remain insufficiently characterized"

"the specificity data obtained for one batch of antibody do not necessarily reflect the properties of another one" Voskuil ( Commercial **antibodies** and their validation. Version 2. F1000Res. 2014 Oct 2 [revised 2014 Oct 15];3:232. doi: 10.12688/f1000research.4966.2. eCollection 2014) describes the relatively unknown practice of some antibody manufacturers to use historical data sheets for antibodies to which they do not apply. This should also be mentioned and cited.

The recommendations in paragraph 4 are commendable. However, if manufacturers are expected to carry this out on every lot they sell, the author must acknowledge that the cost of antibodies will have to increase. 

"As proposed by others ( Bradbury & Pluckthun, 2015) end-users should consider boycotting companies not complying with this demand, or at least stay away from products lacking a proper lot-specific documentation." Actually, this was not our main proposal. Our main point (amplified in Getting to reproducible antibodies: the rationale for sequenced recombinant characterized reagents. **Bradbury** AR, Plückthun A. Protein Eng Des Sel. 2015 Oct;28(10):303-5. doi: 10.1093/protein/gzv051) was that we should move away from the use of polyclonal antibodies altogether and use only well characterized sequenced recombinant antibodies. Only in this way can we ensure antibody reproducibility. 

In the last paragraph, the author indicates that recombinant antibodies (or other proteins) may solve this problem. However, this will only be the case if such binders can be unequivocally identified, which will only occur if sequences can be referred to unambiguously. Otherwise, as antibody companies are bought and sold, catalog numbers will change and it may become difficult to reproduce experiments, because it will not be clear which original antibody was used.

I have read this submission. I believe that I have an appropriate level of expertise to confirm that it is of an acceptable scientific standard, however I have significant reservations, as outlined above.

# Response

**Published:** 2015-11-12

**Authors:** Albert Jeltsch

**Affiliations:** Uni Stuttgart, Germany

- *“Paragraph 2 should also indicate that antibodies purportedly recognizing PTMs at specific sites, need to have their recognition specificity also tested against the same PTM at different sites, in the same protein, or others, and with the same core sequence or others. Many so-called specific phosphotyrosine antibodies actually recognize the phosphotyrosine modification independently of its sequence context.”*
  Reply: We agree. This point was mentioned in paragraph 2 on p. 2 using methylation at H3K9 and H3K27 as an example. “Another difficulty is that some histone modifications such as methylation or acetylation of H3K9 and H3K27 lie within an identical amino acid context (ARKS motif), which makes the readout of the target peptide sequence outside of this central motif very important as well.” We have modified this paragraph to make the point clearer and better reflect what the reviewer was asking for: “Moreover, the antibody should bind the modified amino acid residue only at defined modification sites on the target protein, which implies that not only the modification but also the amino acid sequence must be recognized. This is particularly difficult for some histone modifications such as methylation or acetylation of H3K9 and H3K27 which occur within an identical amino acid context (ARKS motif) and make the readout of the target peptide sequence outside of this central motif vital as well.”
- *"In spite of the intricate task of producing histone modification antibodies and their crucial role in chromatin biology, surprisingly, they remain to be insufficiently characterized" should be changed to "In spite of the intricate task of producing histone modification antibodies and their crucial role in chromatin biology, surprisingly, they remain insufficiently characterized"*
  Reply: This has been changed as proposed.
- *"the specificity data obtained for one batch of antibody do not necessarily reflect the properties of another one" Voskuil (Commercial antibodies and their validation. Version 2. F1000Res. 2014 Oct 2 [revised 2014 Oct 15];3:232. doi: 10.12688/f1000research.4966.2. eCollection 2014) describes the relatively unknown practice of some antibody manufacturers to use historical data sheets for antibodies to which they do not apply. This should also be mentioned and cited.*
  Reply: Thank you. This point and reference has been added.
- *“The recommendations in paragraph 4 are commendable. However, if manufacturers are expected to carry this out on every lot they sell, the author must acknowledge that the cost of antibodies will have to increase.”*
  Reply: Please note in the original manuscript on p. 2 we stated that a shift to sustainable reagents (i.e. away from polyclonal antibodies) “would help to reduce the necessary financial and workload efforts associated with quality control of polyclonal antibodies and ensure sustainability”, which partially addressed this point. We now added an additional sentence stating “While one may expect that better quality control will increase the prices of commercial antibodies, end-customers will not be forced to conduct their own quality control and they will not waste money for non-functional antibodies, so that the overall final costs may not be much higher.” to incorporate this request more explicitly.
- "As proposed by others (Bradbury & Pluckthun, 2015) end-users should consider boycotting companies not complying with this demand, or at least stay away from products lacking a proper lot-specific documentation." Actually, this was not our main proposal. Our main point (amplified in Getting to reproducible antibodies: the rationale for sequenced recombinant characterized reagents. Bradbury AR, Plückthun A. Protein Eng Des Sel. 2015 Oct;28(10):303-5. doi: 10.1093/protein/gzv051) was that we should move away from the use of polyclonal antibodies altogether and use only well characterized sequenced recombinant antibodies. Only in this way can we ensure antibody reproducibility.”
  Reply: We have added the citation to this very insightful paper now also at the corresponding place in our manuscript and also added the Prot. Eng. Des. Sel. reference. “As already proposed, in a long-term perspective, a shift away from polyclonal antibodies towards alternative reagents, which can be produced at constant quality, would help to reduce the necessary financial and workload efforts associated with quality control of polyclonal antibodies and ensure sustainability (Bradbury & Plückthun, 2015, Nature 518, 27-29; Bradbury & Plückthun, 2015, Prot. Eng. Des. Sel. 28, 303-305).”
- *“In the last paragraph, the author indicates that recombinant antibodies (or other proteins) may solve this problem. However, this will only be the case if such binders can be unequivocally identified, which will only occur if sequences can be referred to unambiguously. Otherwise, as antibody companies are bought and sold, catalog numbers will change and it may become difficult to reproduce experiments, because it will not be clear which original antibody was used.”*
  Reply: This is a valid point. We have added on sentence to stress this: “The recombinant reagents would be particularly promising, because their sequences can be published, which ensures full transparency and reproducibility.”
